# Adoption and Optimization of Genomic Selection To Sustain Breeding for Apricot Fruit Quality

**DOI:** 10.1534/g3.120.401452

**Published:** 2020-10-16

**Authors:** Mariem Nsibi, Barbara Gouble, Sylvie Bureau, Timothée Flutre, Christopher Sauvage, Jean-Marc Audergon, Jean-Luc Regnard

**Affiliations:** * INRAE, Génétique et Amélioration des Fruits et Légumes, 84143 Montfavet Cedex, France; † INRAE, Avignon University, UMR SQPOV, 84914 Avignon, France; ‡ INRAE, CNRS, AgroParisTech, Univ. Paris-Saclay, GQE-Le Moulon, 91190 Gif-sur-Yvette, France; § Université Paris-Saclay, INRAE, CNRS, AgroParisTech, GQE - Le Moulon, 91190, Gif-sur-Yvette, France

**Keywords:** *Prunus armeniaca*, Genomic, Prediction, Accuracy, Genetic architecture, Multivariate modeling, GenPred, Shared data resources

## Abstract

Genomic selection (GS) is a breeding approach which exploits genome-wide information and whose unprecedented success has shaped several animal and plant breeding schemes through delivering their genetic progress. This is the first study assessing the potential of GS in apricot (*Prunus armeniaca*) to enhance postharvest fruit quality attributes. Genomic predictions were based on a F1 pseudo-testcross population, comprising 153 individuals with contrasting fruit quality traits. They were phenotyped for physical and biochemical fruit metrics in contrasting climatic conditions over two years. Prediction accuracy (PA) varied from 0.31 for glucose content with the Bayesian LASSO (BL) to 0.78 for ethylene production with RR-BLUP, which yielded the most accurate predictions in comparison to Bayesian models and only 10% out of 61,030 SNPs were sufficient to reach accurate predictions. Useful insights were provided on the genetic architecture of apricot fruit quality whose integration in prediction models improved their performance, notably for traits governed by major QTL. Furthermore, multivariate modeling yielded promising outcomes in terms of PA within training partitions partially phenotyped for target traits. This provides a useful framework for the implementation of indirect selection based on easy-to-measure traits. Thus, we highlighted the main levers to take into account for the implementation of GS for fruit quality in apricot, but also to improve the genetic gain in perennial species.

Apricot (*Prunus armeniaca*) is a perennial fruit crop pertaining to Rosaceae family and *Prunus* genus, which encompasses several economically important species such as peach, almond, cherry and plum. It is one of the leading stone fruit species due to its economic contribution to the fruit industry. From a biological standpoint, apricot is characterized by its diploid genome (2n = 2x = 16) of 294 Mb/1n and its high heterozygosity (Arumuganathan and Earle 1991). The availability of a high-quality genome sequence in peach, defined as a reference *Prunus* species highly genetically characterized (Infante *et al.* 2008; Verde *et al.* 2013), as well as the high level of synteny between the *Prunus* species, have paved the way for elucidating the genetics of key commercial traits in *Prunus* species (Aranzana *et al.* 2019). They provide both a powerful framework for apricot genetic improvement and valuable tools to elucidate the genetic architecture of traits of interest.

Since the sixties, apricot breeding programs have been geared toward conventional breeding based on mass field selection, a time-consuming and labor-intensive process, which might reach 15 to 20 years from pre-breeding to the release of a new variety. Besides the length of apricot breeding cycle, several biological features inherent to this species impede genetic progress such as its wide range heterozygosity and a preferential self-incompatibility regime that induces uneven production according to climatic conditions. Recently, a particular focus has been projected toward fruit quality, a dynamic concept which encompasses a broad range of attributes linked to attractiveness, flavor, taste and texture with reference to fruit color, balance of sugars and acids and shelf-life. The burgeoning interest in fruit quality aims at shaping a sustainable fruit industry taking into account consumer preference trends that are expressed in a competitive landscape faced with climate change. Furthermore, commercial depreciation due to ripeness deficiencies resulting from early harvest and susceptibility to flesh mealiness incented the breeders to circumvent the issues linked to postharvest quality and thus contribute to the enhancement of fruit quality metrics (Gatti *et al.* 2009). In the scope of intrinsic challenges of apricot, fruit quality-oriented selection schemes aim to meet consumers’ needs for improved quality attributes and address stakeholders’ demands in the apricot sector. Therefore, controlled cross-pollination schemes allow recombination of desirable characteristics according to the integrated concept of ideotype, which is likely to guide biological designs of improved varieties through the identification and the integration of causal variants for high-value traits as well as the elimination of deficiencies (Donald 1968; Ramstein *et al.* 2019).

Here emerges one of the prominent impetuses of marker-based breeding approaches, a breeding strategy whose feasibility strongly tailored by the genetic architecture of target traits. Indeed, marker-assisted selection (MAS), is particularly relevant for monogenic inheritance, while genomic selection (GS), a promising breeding approach that has revolutionized animal and plant breeding communities, is favored for oligogenic and polygenic inheritance (Kumar *et al.* 2012). GS is likely to capture the missing heritability of complex traits by modeling thousands of single nucleotide polymorphisms concomitantly (Makowsky *et al.* 2011; Resende *et al.* 2012a). Meuwissen *et al.*’s landmark article (2001) laid the foundation for predicting genetic merit in plant and animal breeding and thus identifying superior genotypes among selection candidates according to their whole-genome sequence information (de los Campos *et al.* 2013). Unlike MAS, which pinpoints putative genes underlying the traits of interest, GS potentially considers all markers’ effects without prior selection (Makowsky *et al.* 2011; Resende *et al.* 2012a). This breeding approach is at its outset for crop plants and notably for perennial trees that are characterized by long breeding cycles due to the length of juvenile phase and generation time. Therefore, the recourse to GS for perennial species arises from the need to accelerate the pace of the breeding process. The relevance of this breeding approach has been assessed in forest trees such as eucalyptus (Resende *et al.* 2012a), black spruce (Lenz *et al.* 2017), white spruce, loblolly pine (Resende *et al.* 2012b), maritime pine (Bartholomé *et al.* 2016) as well as in perennial fruit crops such as grapevine (Fodor *et al.* 2014), apple (Muranty *et al.* 2015), citrus (Minamikawa *et al.* 2017), cranberry (Covarrubias-Pazaran *et al.* 2018) and kiwifruit (Testolin 2011).

Large-scale genomic information against limited phenotypic records leads to an ascertainment bias due to the number of predictors (p), which is higher than the number of observations (n), resulting in multicollinearity and overfitting and accordingly low prediction performance (Desta and Ortiz 2014). To alleviate this statistical challenge due to dimensionality, a wide range of mathematical models are intended to infer linear combinations of the original predictors in order to reduce through shrinking regression coefficients back toward zero (Whittaker *et al.* 2000; Gianola *et al.* 2003; Solberg *et al.* 2009). The extent of shrinkage of the marker effects differ across prediction models. For instance, in ridge regression shrinkage is performed equally across markers. However, this assumption is likely to be unreal because some markers are in linkage disequilibrium (LD) with loci with no genetic variance (Goddard and Hayes 2007). Conversely, in models designed under the Bayesian framework, shrinkage of effects is marker-specific (Crossa *et al.* 2017). Further, the performance of GS is markedly influenced by several factors including marker density (Grattapaglia and Resende 2011; Lenz *et al.* 2017), training population size (Grattapaglia and Resende 2011), genetic relationship between training population and breeding population (Rincent *et al.* 2012; Isidro *et al.* 2015; Lenz *et al.* 2017), population structure (Zhong *et al.* 2009; Rincent *et al.* 2017), the extent of LD (Daetwyler *et al.* 2008; Wientjes *et al.* 2013; Liu *et al.* 2015), statistical models (Lorenzana and Bernardo 2009; Heslot *et al.* 2012; Resende *et al.* 2012b; Onogi 2020), trait heritability (Calus *et al.* 2008) as well as the genetic architecture of target traits (Daetwyler *et al.* 2010; Morgante *et al.* 2018). Along with the ideotype concept, multiple traits of interest can also be considered simultaneously through multivariate models to achieve more accurate predictions in comparison to single-trait models. Several simulation and empirical studies shed light upon the significant potential of multiple trait genomic prediction in optimizing prediction performance (Calus and Veerkamp 2011; Guo *et al.* 2014; Covarrubias-Pazaran *et al.* 2018; Karaman *et al.* 2018; Michel *et al.* 2018). In this regard, the selection index strategy permits breeders to obtain genotypes that concomitantly incorporate several desirable characteristics. However, the efficiency of selection for multiple traits simultaneously depends considerably on the genetic correlation between these traits, that reflects the extent to which selection for a focal trait triggers an indirect response to selection for a secondary trait (Akdemir *et al.* 2019; Rana *et al.* 2019). In conjunction with the optimization of prediction model design, the integration of the insights gained by elucidating the genetic architecture of traits under selection might be of great interest. Several studies have emphasized the potential of including genomic information underlying the variation of target traits in prediction models (Spindel *et al.* 2016; Fang *et al.* 2017; Lopes *et al.* 2017; Liu *et al.* 2019).

Therefore, the main objectives of our study were to (1) gain further insights into key fruit quality traits which are difficult to access, (2) evaluate the performance of GS prediction model applied to breeding for apricot fruit quality and (3) optimize GS accuracy by accounting for QTL mapping findings in prediction models and performing predictions under a multivariate framework.

## Materials and Methods

### Plant material

The plant material used in this study is a F1 pseudo-testcross progeny of 153 individuals issued from a cross between ‘Goldrich’ and ‘Moniqui’ cultivars, which exhibit contrasted fruit quality traits. ‘Goldrich’ cv., used as female parent, is a North American early-season apricot cultivar. Self-incompatible, it is characterized by large, firm, orange fruit without blush and with a high level of acidity (Muñoz-Sanz *et al.* 2017). ‘Moniqui’ cv., used as male parent, is a Spanish season apricot cultivar. Self-incompatible, it is characterized by large, soft and tasty white flesh fruit. ‘Moniqui’ is characterized by a high ethylene production, which results in a fast evolution at maturity and post-harvest, while ‘Goldrich’ presents a lower ethylene production, which results in an average fruit evolution at maturity and post-harvest. The F1 progeny was grown at the INRAE experimental field of Amarine in southern France. Seedlings were randomly planted on their own roots in 2005. Trees were managed under integrated management system which implies that orchards are geared toward a sustainable production system with a trend to reduce the use of phytosanitary products.

### Phenotyping for fruit quality

The phenotypic characterization of the Goldrich × Moniqui (Go×Mo) progeny was carried out over two consecutive years 2006 and 2007, which showed contrasted climatic conditions for 10 quality parameters of agronomic interest. These traits refer to the criteria which underpin consumers’ perception of apricot fruit and meet the exigencies of stakeholders in the apricot sector.

A total of 40 fruits per genotype were randomly collected close to physiological maturity stage. Fruits were sorted according to their global firmness, determined by the pressure (kPa) required to achieve 3% deformation of fruit height with a multipurpose texture analyzer (Pénélaup, Serisud, Montpellier, France). Fruits were subdivided into three homogenous lots of four fruits per genotype of contrasting maturity: commercial maturity stage with pressure from 130 to 80 kPa, half ripe stage from 80 to 50 kPa and mature fruits with firmness less than 50 kPa. The physical, physiological and biochemical traits were measured on these three representative batches for all genotype. The fruit weight (g) was measured at the same time as firmness. The ground color (Hue.g) of the non-blushed side (unexposed to sunlight) was determined using a CR-400 chromameter (Minolta, Osaka, Japan) and expressed in the CIE 1976 L*a*b*color space (illuminant D65, 0° view angle, illumination area diameter 8 mm). Hue angle, was computed using the chromaticity coordinates $a^{*}$ and $b^{*}$ as follows:

Hue=tg−1(b*a*) (1)

The ethylene production rate was assessed as physiological parameter linked to maturity stage of climacteric fruits. Ethylene production, expressed in nmol ${\text{kg}}^{-1}{\text{h}}^{-1}$, was measured by gas chromatography after 1 h of confinement in a hermetically closed jar (Chambroy *et al.* 1995; Bureau *et al.* 2009). Then, flesh color was measured and fruits were cut and frozen at -20° for further biochemical analyses. Fruit stones were weighed individually (kg). Fruit pieces were ground with an Ultra-Turrax T25 equipment (Ika Labortechnik, Staufen, Germany) to obtain a slurry. The refractive index (RI) which stands for the solid soluble content (SSC) was determined with a digital refractometer (PR-101 ATAGO, Norfolk, VA) and expressed in °Brix at 20°. Titratable acidity (TA) was determined by neutralization up to pH 8.1 with 0.1 N NaOH and expressed in meq 100 g^-1^ of fresh weight using an autotitrator (Methrom, Herisau, Switzerland). Soluble sugars (glucose, fructose, sucrose) and organic acids (malic acid and citric acid) were quantified using an enzymatic method using kits for food analysis (Boehringer Mannheim Co., Mannheim, Germany) and expressed in g 100${\text{g}}^{-1}$of fresh weight for sugars and meq 100${\text{g}}^{-1}$of fresh weight for acids. These measurements were performed with an automatic analyzer BM-704 (Hitachi, Tokyo, Japan).

### Statistical modeling of the phenotypic data

Statistical modeling of the fruit quality attributes was performed using R software version 4.0.2 (R Core Team 2020). Significance assessment of variance components was carried out using ANOVA tests to determine the significant factors contributing to the phenotypic variation intended to be included in the adjustment model. In light of significance tests outcome, phenotypic data were adjusted using ‘lmer’ function provided in lme4 package (Bates *et al.* 2015) within a mixed model framework:

$$y_{ijk}=µ+\alpha_{i}+\beta_{j}+\delta_{k}+\alpha \beta_{ij}+e_{ijk}$$ (2)

In equation 2, $y_{ijk}$ is the phenotypic value of the genotype i for the year j and the maturity group k, µ is the overall mean, $\alpha_{i}$ is the random effect of the genotype i, $\beta_{j}$ refers to the fixed effect of the year j, $\delta_{k}$ is the fixed effect of the maturity group k corresponding to the fruit lot, $\alpha \beta_{ij}$ is the interaction effect of the genotype i and the year j and $e_{ijk}$ is the random residual effect.

### Heritability computation

Broad-sense heritability $H^{2}$ for fruit quality traits, defined as the proportion of phenotypic variance attributed to additive, dominance and epistatic patterns, was computed using equation 3:

$$H^{2}=\frac{\sigma_{g}^{2}}{\sigma_{g}^{2}+\frac{\sigma_{gy}^{2}}{n_{y}}+\frac{\sigma_{l}^{2}}{n_{l}}+\frac{\sigma_{y}^{2}}{n_{y}}+\frac{\sigma_{e}^{2}}{n_{y}*n_{l}}}$$ (3)

where $\sigma_{g}^{2}$ is the genetic variance, $\sigma_{gy}^{2}$ is the variance attributed to the interaction between genotypes and years, $n_{y}$ refers to the number of years and $n_{l}$ is the number of fruit lots.

### Genotyping data

Genotyping by sequencing, performed according to the protocol described by Elshire *et al.* (2011), was carried out within the FruitSelGen project. Regarding the high level of synteny between the apricot and peach genomes, the fastq sequences were aligned to the peach genome (Verde *et al.* 2013). Raw data filtering, sequence alignment and variant calling were performed using GATK software (Genome Analysis Toolkit) (McKenna *et al.* 2010). The outcome of a further filtering process using VariantAnnotation package (Obenchain *et al.* 2014) resulted in a set of 61,030 SNPs with a genotype quality score greater than 20 and a missing rate lower than 5%. Out of the 184 individuals, 31 individuals exhibiting spurious genotypic profile were discarded and thus 153 individuals were kept for downstream analysis. SNP markers were coded as 0, 1 and 2, according to the number of copies of the alternative allele and missing marker information was imputed as the mean of the genotypic scores of non-missing data at the level of each maker.

### Construction of the linkage maps

The advent of genomic selection to breed for apricot quality traits requires a better understanding of their genetic architecture. However, up to now, the genetic determinism underlying fruit quality in apricot was scarcely investigated (Ruiz *et al.* 2010). Thus, linkage mapping was performed in order to uncover the genetic architecture of the 10 fruit quality traits. Prior to QTL identification, two genetic linkage maps were constructed for the full-sib progeny using a pseudo-test cross mapping strategy (Grattapaglia and Sederoff 1994). The whole set of 61,030 SNPs was filtered according to Mendelian inheritance, and those presenting strong deviation from Hardy-Weinberg equilibrium (p-value < 1 × $10^{-6}$) were discarded using the function filterSegreg provided by rutilstimflutre package (Flutre 2015). Afterward, the markers which depicted more than 1% of missing information and more than 1% of genotyping errors were eliminated and linkage group (LG) clustering, marker ordering and genetic distance calculations were achieved by means of mstmap.data.frame function under ASMap package (Taylor and Butler 2017). Maps construction was performed using Kosambi’s mapping function and a logarithm of the odds ratio (LOD) of 3.

### QTL detection

In order to provide insights into the genetic architecture of the target traits, we performed a composite interval mapping strategy using R/qtl package (Broman *et al.* 2003) with the aim of identifying the genomic regions underpinning apricot fruit quality. In this respect, 1,000 permutations were undertaken with a significance level set at 0.01 in order to identify putative QTL and determine the threshold of LOD scores. Then, the part of phenotypic variance explained by SNPs significantly linked to target traits was estimated. Additionally, a joint QTL detection analysis was performed on two independent datasets recorded in 2006 and 2007 with the aim of assessing the stability of QTL associated to the adjusted means corresponding to the phenotypic records. The graphical representation of the two genetic maps as well as QTL-linked markers was drawn using MapChart 2.3 software (Voorrips 2002).

### Univariate genomic prediction modeling

Prediction of the genomic estimated breeding values was performed using a baseline model where the genomic information as well as the phenotypic records were fitted in order to estimate marker effects and thus the breeding values:

y=Xβ+Zu+e (4)

In equation 4, *y* is the vector of the phenotypic records, *X* is an incidence matrix for fixed effects relating fruit quality to the vector of fixed effects *β*, *β* is a vector of fixed effects estimates, *Z* is an incidence matrix for random effects relating fruit quality to the vector of random additive genetic effects *u*. *Z* is inferred from SNPs’ allelic dosage for each individual coded as 0, 1 and 2 according to the number of copies of the minor allele. The term *u* is the vector of random effects and *e* denotes the vector of random errors.

Random SNP effects were assumed to follow a normal distribution *u ∼ N* (*0*, *I*$\sigma_{u}^{2}$) and random residual effects a normal distribution *e ∼ N* (*0*, *I*$\sigma_{e}^{2}$) with I is the identity matrix, $\sigma_{u}^{2}$ is variance of random effects and $\sigma_{e}^{2}$ residual variance. GEBVs were computed as the sum of estimated marker effects multiplied by the corresponding allelic doses, as follows:

$$GEBV_{i}=\overset{n}{\underset{j=1}{\sum }}Z_{ij}^{’}{\hat{u}}_{j}$$ (5)

In equation 5, $Z_{ij}^{’}$ denotes the matrix of allelic doses for the $i^{th}$ individual in the validation partition at the $j^{th}$ locus and ${\hat{u}}_{j}$ is the estimated effect at the $j^{th}$ locus.

### Cross-validation procedure

The performance of prediction, mirrored in the predictive accuracy for the 10 key quality traits, was assessed using a cross-validation strategy where data were randomly partitioned into two subsets: 75% of the reference set was assigned to the training set intended to calibrate the prediction model and the remaining 25% was used as the validation set whose phenotypes were assumed to be unknown. This cross-validation scheme was iterated 100 times where samples were drawn with replacement from the reference set. Pearson’s correlation between predicted phenotypes and the observed ones was used to determine the accuracy of the predicted phenotypes.

### Factors controlling genomic prediction accuracy (PA)

As several parameters control the prediction performance such as statistical models, training population size and marker density, these parameters were investigated using randomly drawn subsets of the reference dataset in order to point out the factors governing the potential variation in PA, to assess their respective effect.

#### Impact of statistical prediction models:

Within the framework of genomic prediction, various statistical methods have been proposed in literature. These models share the same prediction equation for the estimation of the GEBVs while they are grounded on different assumptions concerning markers effects. Five Bayesian models were explored: Ridge regression best linear unbiased prediction (RR-BLUP) model implemented in the rrBLUP package (Endelman 2011) as well as Bayes A, Bayes B, Bayes C, Bayesian LASSO (BL) and Bayesian ridge regression (BRR) implemented in the package BGLR (Pérez and de los Campos 2014).

Genomic prediction models vary in their assumptions regarding marker effects. RR-BLUP model postulates that all SNPs have identical variance with small but non-zero effect. All marker effects are homogeneously shrunk toward zero but markers are allowed to have unequal effects (Desta and Ortiz 2014). Unlike RR-BLUP, Bayesian models posit that each SNP has its own variance. Under Bayes A model, markers effects follow a normal distribution and variances follow a scaled inverted ${\text{χ}}^{2}$ distribution (Meuwissen *et al.* 2001). Similar to Bayes A, Bayes B yields a scaled inversed ${\text{χ}}^{2}$ with π > 0 so that several SNPs have zero effect. Unlike Bayes A, Bayes B applies both shrinkage and variable selection methods. Bayes A and Bayes B are both characterized by a prior probability (π) that a SNP has zero effect and a probability (1−π) of marker effects that are shrunk toward zero. In Bayes A, all markers have non-zero effect π = 0. On the contrary, Bayes C states that the prior of zero SNP effects π is considered as unknown. Similar to Bayes B model, Bayes C applies both shrinkage and variable selection. But, unlike Bayes B, Bayes C is characterized by a Gaussian distribution (Desta and Ortiz 2014). BRR model, a Bayesian version of Ridge regression, assumes non-zero and normally distributed marker effects and equal marker variances. Similar to RR-BLUP, BRR applies a homogenous shrinkage across markers (de los Campos *et al.* 2013). As for Bayesian LASSO (BL), the Bayesian version of LASSO, it posits a compromise between Lasso and ridge regression (Park and Casella 2008). BL assumes a double exponential distribution of marker variances (Desta and Ortiz 2014).

#### Impact of training population (TP) size:

In order to explore the impact of population size on PA, we used three randomly drawn subsets of 43, 76, and 115 individuals corresponding to 25%, 50% and 75% of the respective study population to elaborate the prediction model and thus compute breeding values of the remaining individuals.

#### Impact of marker density:

Furthermore, we assessed the extent to which randomly selected marker subsets of different sizes, from 1 to 100% could affect the accuracy of prediction.

### Genomic prediction optimization

Herein, we assessed two prediction strategies with a view to the improvement of PA.

#### Accounting for genetic architecture:

The first optimization scenario made use of the information brought by QTL mapping. Thus, SNPs tightly linked to QTL with medium to large effects were included in the prediction models as fixed covariates in order to assess the PA. The genomic prediction model which accounts for prior information on genetic architecture is defined as:

y=X’β’+Z’u’+e (6)

where *y* is the vector of phenotypic observations, *X*’ is the design matrix for fixed effects linking phenotypes to allelic doses of SNPs tagging QTL, β’ is the vector of allelic doses of SNPs closely linked to QTL, *Z*’ is the design matrix for random effects linking phenotypes to the remaining SNPs, u’ is the vector of allelic doses of the remaining SNPs and e is the vector of residuals.

The first phase of analysis was dedicated to QTL detection within the training population that was randomly drawn using 75% of the study population. The identified QTL were subsequently included in genomic prediction models. This procedure was repeated 100 times. The baseline model that includes QTL as fixed-effect covariates attributed different weights to SNPs according to their linkage to QTL. Accordingly, we aimed at assessing the potential of prediction models that treated all SNPs as random and those that placed greater weights on QTL.

#### Multivariate genomic prediction:

A second scenario dedicated to optimizing genomic selection accuracy is the multi-trait prediction implemented using the R package ‘sommer’ (Covarrubias-Pazaran 2016), which provides a framework for fitting multivariate prediction models. Hence, this prediction strategy was performed using Genomic BLUP (GBLUP) model whose equation is provided in equation 7:

$$y_{i}=X_{i}\beta_{i}+Z_{i}u_{i}+e_{i}$$ (7)

where $y_{i}$ is a N × t vector of the phenotypic records for trait i with i = 1, …, t, X is an incidence matrix for fixed effects relating fruit quality to the vector of fixed effects $\beta_{i}$, which is a vector of fixed effects estimates, Z is an incidence matrix for random effects relating fruit quality to vectors of random additive genetic effect $u_{i}$, $u_{i}$ is a vector of random effects and $e_{i}$ denotes the vector of random errors.

The implementation framework of multi-trait models follows that of (Maier *et al.* 2015) and (Covarrubias-Pazaran *et al.* 2018):

$$\begin{array}{l} y_{1}=X_{1}\beta_{1}+Z_{1}u_{1}+e_{1}\;\text{for trait}\;1\\ y_{2}=X_{2}\beta_{2}+Z_{2}u_{2}+e_{2}\text{ for trait}\;2\\ y_{t}=X_{t}\beta_{t}+Z_{t}u_{t}+e_{t}\text{ for trait t} \end{array}$$ (8)

For trait i (i = 1…t), random effects $u_{i}$ and $e_{i}$, fitted within multivariate response model, are assumed to be normally distributed with mean zero with $u∼N(0,A\sigma_{u}^{2}$) and $e∼N(0,I\sigma_{e}^{2}$).

Assuming *A* is the additive genomic relationship matrix computed according to (VanRaden 2008), as:

$$A=WW’/2\sum^{\text{​}}p_{i}(1-p_{i})$$ (9)

In equation 9, *W* is the matrix of marker alleles coded as -1, 0 and 1 for homozygote, heterozygote and homozygote individuals, respectively, $p_{i}$ is the allele frequency of marker i.

Within this context, components of prediction model follow multivariate normal distribution with *y* ∼ MVN (Xβ,V), $u_{i}$ ∼ MVN ($0,\underset{u}{\sum }⊗A$) and $e_{i}$ ∼ MVN ($0,\underset{e}{\sum }⊗R$).

where $\underset{u}{\sum }$is the variance-covariance matrix for marker effects, $\underset{e}{\sum }$ is the variance-covariance matrix for residual effects, ⊗ is Kronecker product of variance-covariance matrices.

$$\text{with}\;\;\;\;\begin{array}{cc} y=\left[\begin{array}{c} y_{1}\\ \begin{array}{c} y_{2}\\ {\begin{array}{c} \ldots \\ y \end{array}}_{t} \end{array} \end{array}\right] & \text{X}=\left[\begin{array}{ccc} X_{1} & \cdots & 0\\ \vdots & \ddots & \vdots \\ 0 & \cdots & X_{t} \end{array}\right] \end{array}$$ (10)

The phenotypic variance covariance matrix (*V*) is denoted as:

$$V=\left[\begin{array}{ccc} Z_{1}K\sigma_{u_{1}{}_{t1}}^{2}Z_{1}^{’}+Z_{1}R\sigma_{e_{t1}}^{2}Z_{1}^{’} & \cdots & Z_{1}K\sigma_{u_{1}{}_{1,t}}Z_{t}^{’}+Z_{t1}R\sigma_{e_{1,t}}Z_{ti}^{’}\\ \vdots & \ddots & \vdots \\ Z_{1}K\sigma_{u_{1}{}_{1,t}}Z_{t}^{’}+Z_{1}R\sigma_{e_{1,t}}Z_{t}^{’} & \cdots & Z_{t}K\sigma_{u_{1}{}_{t}}^{2}Z_{ti}^{’}+Z_{t}R\sigma_{e_{t}}^{2}Z_{t}^{’} \end{array}\right]$$ (11)

where K denotes the covariance matrix for the $K^{th}$ random effect and R = I is an identity matrix for the residual term. The terms $\sigma_{uk_{i}}^{2}$ and $\sigma_{e_{i}}^{2}$ are the genetic and residual variance of trait I, $\sigma_{uk_{ij}}$ and $\sigma_{e_{ij}}$ are the genetic and residual covariance between traits i and j with i = 1,…, t and j = 1, …, t.

Prediction of focal traits within the validation partition was grounded on two multi-trait methods according to the type of proxy predictand as described in Michel *et al.* (2018). Proxies were either phenotypic values or estimated genetic values of the secondary trait.

The second method consisted on combining single-trait predictions into a selection index based on two step method. First, GEBVs of proxies were computed via single-trait baseline model described in equation (4). The vector of GEBVs was, subsequently, included as covariates in the prediction model in order to predict the trait of interest. GEBVs for focal trait are weighted by GEBVs of proxy trait.

$$GEBV_{i}=x_{i}\beta_{proxy}+u_{i}$$ (12)

where $u_{i}$ denotes the random genetic effect of the $i^{th}$ individual, $x_{i}$ is the GEBV for secondary trait being proxy of the $i^{th}$ individual and $\beta_{proxy}$ is the estimated fixed effect of the secondary trait.

These two methods aimed at assessing the performance of multi-trait prediction that hypothetically enables indirect selection for costly and difficult-to-record traits.

In order to further assess the performance of multi-trait models in comparison with single-trait models described in equation (4), missing values were randomly included in the training partition, with an increasing missing rate ranging from 0 to 90% for the focal trait.

### Data availability

Supplemental data are available in Files S1-8. File S1 contains the raw phenotypic data. Descriptive statistics of phenotypic data are presented in File S2 .File S3 contains the estimations of trait heritability. The genotypic data are available in File S4. The results of QTL detection are summarized in File S5 and the two genetic maps are provided in File S6. Pairwise genetic correlation between the traits are available in File S7. R scripts are provided in File S8.

Supplemental material available at figshare: https://doi.org/10.25387/g3.13050107.

## Results

### Exploration of the phenotypic data

**Figure 1:**
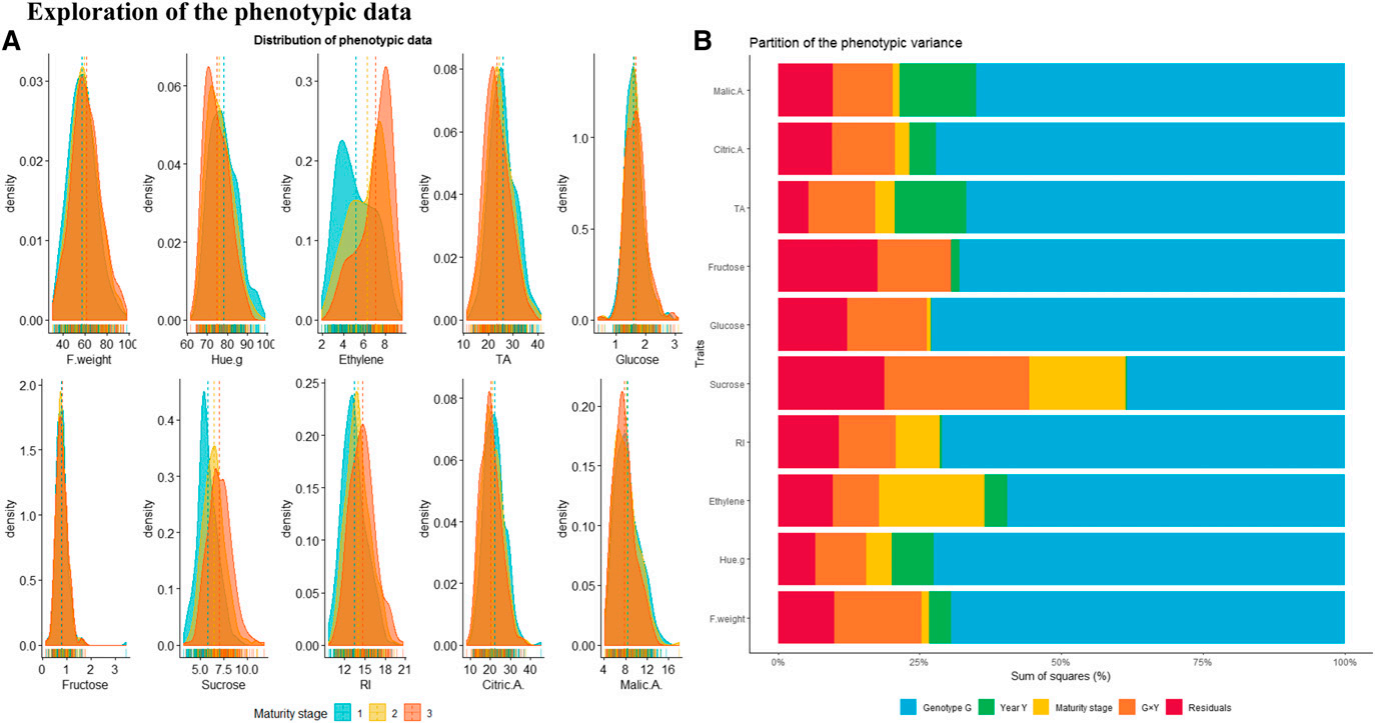
Exploration of the phenotypic data: distribution of phenotypic values for the 10 apricot quality traits in the Go X Mo progeny (A) and components of the phenotypic variation with reference to genotype (G) effect, year (Y) effect, G×Y interaction and fruit stage of maturity (B).

The distribution of phenotypic values according to the maturity stage (Figure 1A) reflects the quantitative determinism of most apricot fruit quality traits, except for ethylene production rate which exhibited a skewed distribution and was adjusted with a logarithmic transformation to restore its normal distribution. Continuous distribution of the phenotypic records points to polygenic inheritance of most traits and a potential contribution of several QTL to the phenotypic variation. The apricot quality traits were positively affected by maturity (Figure 1A). The average phenotypic values for fruit RI and sucrose content increased from 13.7 to 15 °Brix and from 5.5 to 7.0 g 100 g^-1^ of fresh weight respectively, while TA decreased slightly from 25.9 to 21.8 meq 100 g^-1^ fresh weight. A sharp rise in the ethylene production rate was observed throughout ripening, which corresponds to a 94% increase from group 1 to group 3. Likewise, Hue.g changed with maturity stage, the average value of which decreased from 73 to 71 degrees. The range of variability of phenotypic values varied from 8.5% for Hue.g to 30.4% for fructose content. The extent of phenotypic variation reflects the diversity within the genetic pattern of the study population issued from two varieties with contrasted fruit characteristics.

**Figure 2:**
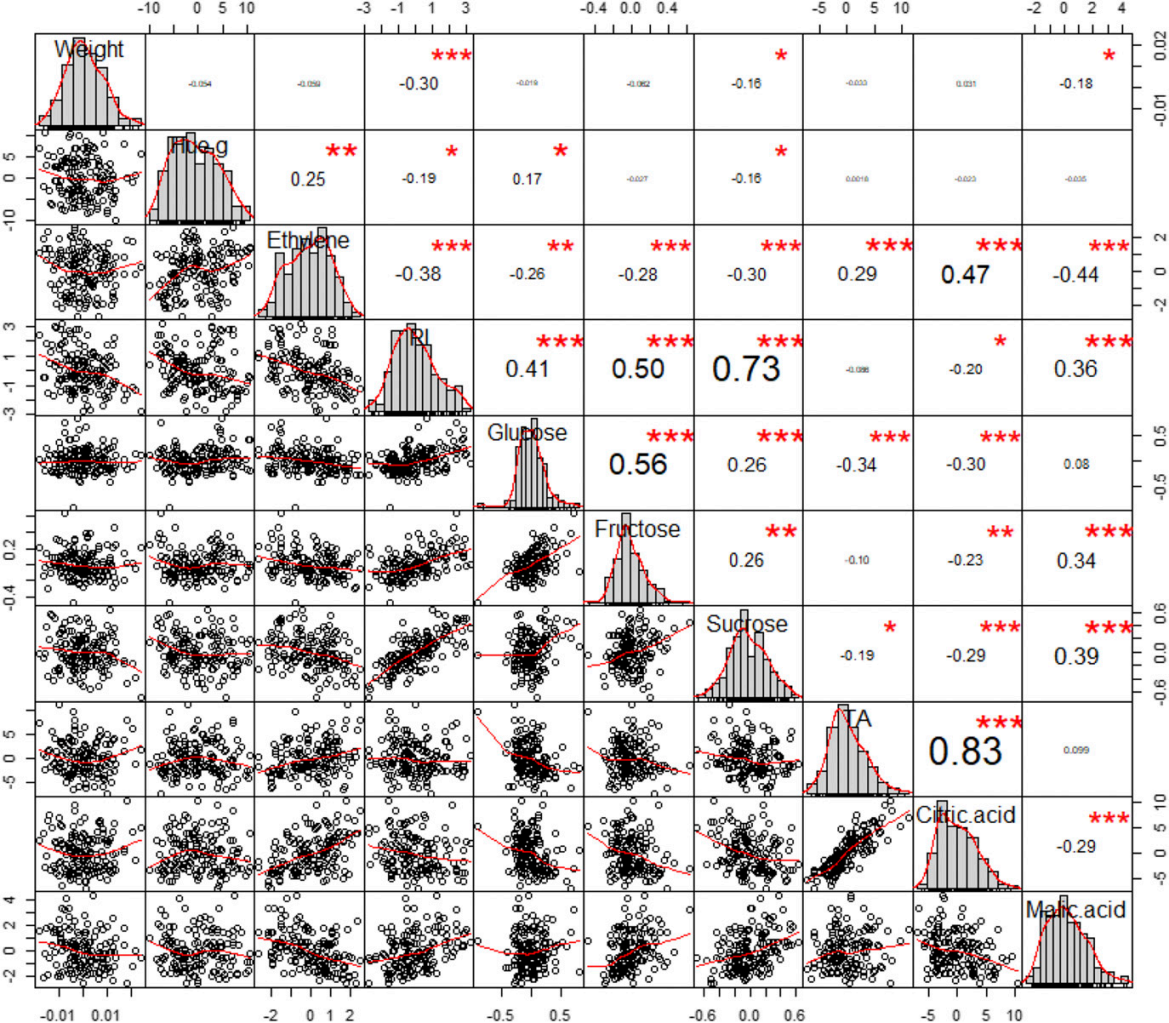
Matrix of pairwise correlations: Bivariate scatterplots (lower off-diagonal) and correlation values between phenotypic values (upper off-diagonal) for 10 apricot fruit quality traits. The distribution of adjusted phenotypic values is shown in the diagonal.

The partition of the phenotypic variance into different sources of variation (Figure 1B) highlights the significant contribution of the year effect (from 0.1% for glucose to 13.5% of the sum of squares for malic acid) as well as the fruit maturity stage modeled by fruit groups (from 0.01% for fructose to 18.6% of the sum of squares for ethylene). The highest contribution to the phenotypic variation is attributed to the genetic pattern reflected in the genotype effect (from 38.4% for sucrose to 72.9% of the sum of squares for glucose) as well as to the interactions between genotype and year (from 8.2% for ethylene to 25.6% of the sum of squares for sucrose). This trend was endorsed by the moderate to high heritability estimates of apricot quality traits (File S3), with broad-sense *H*^2^ ranging from 0.56 for sucrose content to 0.92 for glucose content. Moreover, analysis of apricot fruit quality traits revealed high positive pairwise correlations between TA and Citric.A (r = 0.83), RI and Sucrose (0.73), Glucose and Fructose (r = 0.56), RI and Fructose (r = 0.50) and Ethylene production and Citric.A. (r = 0.47) and Ethylene production and Malic.A. (r = -0.44) (Figure 2).

### Linkage map construction

The linkage mapping for apricot fruit quality was performed according to a pseudo-testcross mapping strategy. Beforehand, markers filtering was implemented according to Mendelian segregation leading to a final set of 4,922 SNPs, of which 2,311 were heterozygous for Goldrich and 1,395 were heterozygous for Moniqui. Then, we applied a stringent quality control by removing markers presenting more than 1% of genotyping error. A total of 366 SNPs was retained for Goldrich and 250 SNPs for Moniqui. Hence, two parental linkage maps were generated. SNPs mapped for Goldrich were distributed on eight LGs, which present an overall length of 562 cM and an average distance of 1.6 cM between adjacent SNPs. For the male parent Moniqui, the genetic map spanned an overall length of 842.3 cM with an average spacing of 3.5 cM between SNPs. The 250 SNPs mapped for Moniqui were positioned on 10 LGs, where chromosomes 1 and 7 were split into two LGs each.

### QTL detection

**Figure 3:**
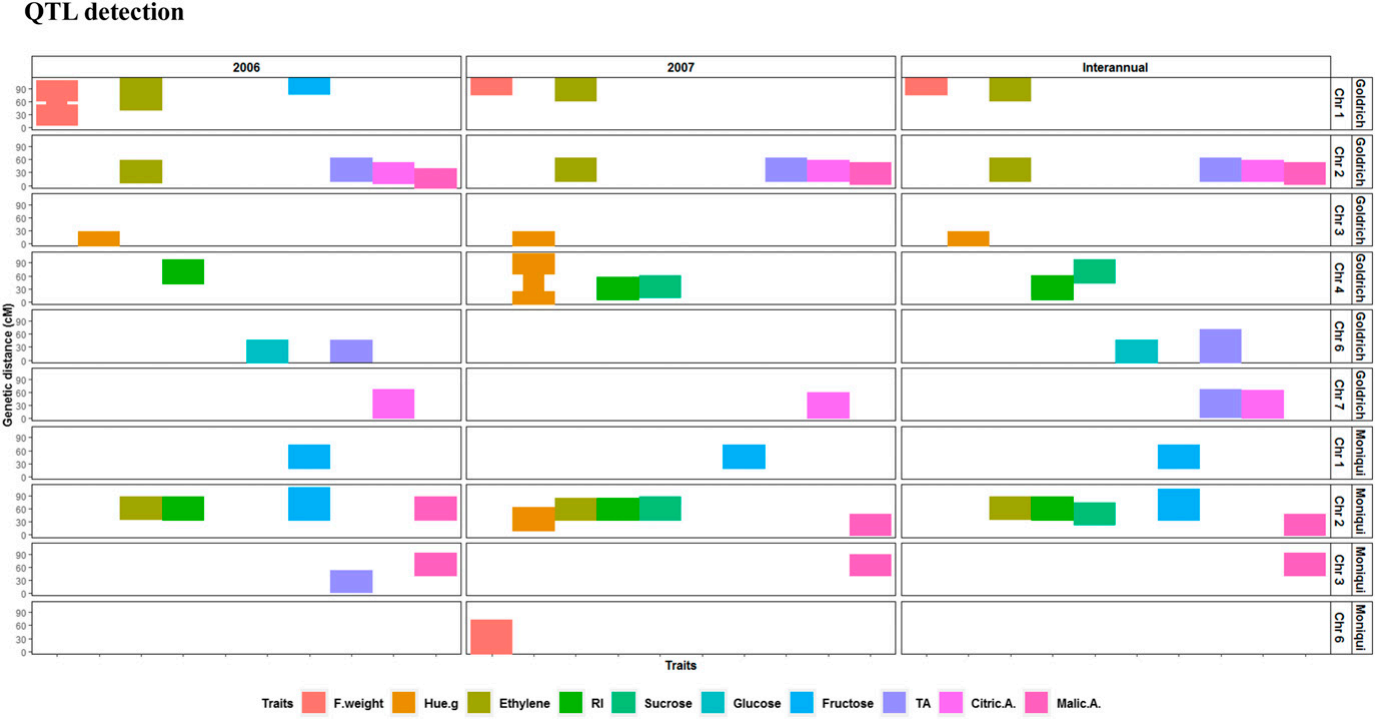
QTL detection performed on adjusted phenotypic values of 10 apricot fruit quality traits according to a pseudo-test cross mapping strategy where two genetic linkage maps were constructed for Goldrich and Moniqui. A joint linkage analysis was carried out across years on two independent datasets recorded in 2006 and 2007. On the y axis, positions of SNPs significantly linked to targeted traits expressed in cM. On the x axis, traits distributed across years and genetic backgrounds. Only chromosomes enclosing significant SNPs are represented.

The linkage analysis was performed using BLUPs. The across-years analysis undertaken using composite interval mapping revealed 20 significant QTL spread over all LGs except LGs 5 and 8 (Figure 3), which explained from 7.6% (TA) to 51.2% (Hue.g) of the observed phenotypic variance and whose peak LOD values varied from 3.44 (TA) to 23.8 (Hue.g) for the 10 fruit quality traits (Table S4). Two major QTL, that explain 23.1% and 21.6% of the phenotypic variability, were detected for RI. One major QTL explaining 51.2% of the observed variability was found for Hue.g, one major QTL explaining 43.7% for Ethylene, one major QTL explaining 24.6% for Citric.A and one major QTL explaining 22.9% for Sucrose.

With reference to the annual linkage analyses, 19 QTL were detected for apricot fruit quality in 2006 and 19 QTL in 2007. Seven QTL showed stability across years, being consistently detected in 2006 and in 2007. The amount of explained variance ranged from 7.9% (Ethylene) to 46.1% (Hue.g) for 2006 and from 0.5% (Fruit weight) to 46.7% (Hue.g) for 2007, while LOD values varied from 3.3 (TA) to 20.5 (Hue.g) for 2006 and from 3.5 (TA) to 20.9 (Hue.g) for 2007. Detailed information is provided in Files S5 and S6.

In terms of colocalization, QTL for Sucrose coincided with QTL for RI on LG2 for Moniqui and on LG4 for Goldrich, while QTL for TA coincided with QTL for Citric.A on LG7 for Moniqui, with QTL for Glucose on LG6 for Goldrich and with Ethylene on LG2 for Goldrich (Figure 3). QTL for RI and Ethylene colocalized on LG2 for Moniqui. In addition, QTL intervals for Malic.A, Sucrose, Fructose, RI, Ethylene and Hue.g presented an overlap on LG 2 of Moniqui parental linkage map. Finally, QTL associated to Glucose and TA coincided on LG6, while those for Fructose, Ethylene and F.weight overlapped on LG1 of Goldrich parental linkage map.

### Genomic prediction accuracy

**Figure 4:**
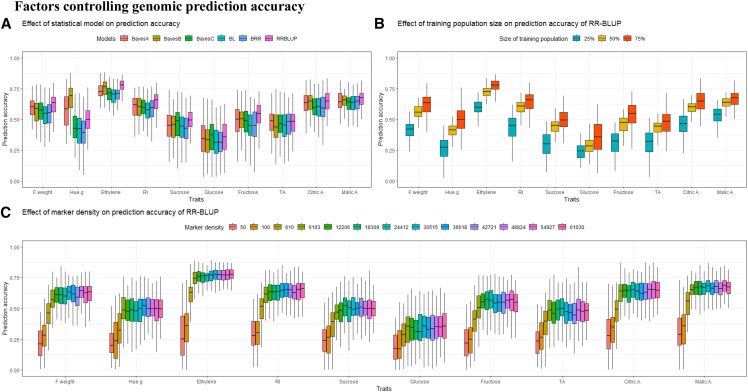
Variation in accuracy of genomic prediction for ten apricot fruit quality traits using a random cross-validation (100 replicates). Effect of the GS statistical model: RR-BLUP, Bayes A, Bayes B, Bayes C, Bayesian LASSO and BRR (A); Effect of the training population size using subsets of 43, 76, and 115 individuals randomly drawn corresponding to 25%, 50% and 75% of the study population using RR-BLUP model (B). Effect of marker density using randomly selected SNP subsets: 50, 100 and from 1 to 100% out of 61,030 SNPs using RR-BLUP model.

The GS accuracy was assessed using different statistical models, different sizes of training population and different subsets of markers randomly distributed along the genome. Results are provided in Figure 4.

### Factors controlling genomic prediction accuracy

#### Impact of statistical prediction models:

We investigated prediction performance for six models (RR-BLUP, Bayes A, Bayes B, Bayes C, BL and BRR). Across the traits, the overall average PA varied from 0.31 with BL (for Glucose) to 0.78 with RR-BLUP (for Ethylene) (Figure 4A). RR-BLUP outperformed Bayes A, Bayes B, Bayes C, BL and BRR for six traits out of 10 (F.weight, Ethylene, RI, Sucrose, Fructose and Malic.A). For three traits (Glucose, TA and Citric.A) similar PA was yielded using RR-BLUP and Bayes A, while Bayes B exhibited the best prediction performance for Hue.g. BL, BRR and Bayes C models provided similar prediction performance across all traits. Among the 10 apricot fruit quality traits, Glucose and TA displayed the lowest average PA regardless of the investigated model.

To further explore the impact of statistical models on prediction performance, we assessed the magnitude of variation appraised by standard deviation of PA of the tested models. Averaged over 10 traits, RR-BLUP showed the lowest variation in PA (0.09) and BRR the highest (0.11). The lowest variation in PA was noted for Ethylene (0.05) and Citric.A (0.09) and the highest variation for Glucose (0.15) and Fructose (0.14).

Then, assessment of variation in PA according to factors such as marker density and training population size was performed with RR-BLUP model, which represents an optimal compromise between prediction performance and computational time.

#### Impact of training population (TP) size:

To assess the impact of TP size on accuracy, we performed genomic prediction using 43, 76 and 115 individuals. As shown in Figure 4B, the increase in TP size resulted in a substantial increase in PA, which ranged from 11 to 24% as a response to the increase in TP size from 25 to 75%.

#### Impact of the number of markers:

PA increased with the number of markers, regardless of the trait genetic architecture, and became steady reaching a plateau at about 6,103 SNPs corresponding to 10% of the total number of markers (Figure 4C). No significant improvement in accuracy was noted when more than 6,103 SNPs were used and with only a rather small number of markers, the model was able to accurately predict the phenotypes in the validation set. Conversely, the average PA dropped from 0.55 to 0.25 across traits when the number of markers used in the prediction model dropped from 6,103 to 100, and the drop was steeper when the number of SNPs was below 50. In addition, decreasing the number of markers resulted in an increase in the variability of PA for all the traits under investigation. For instance, for ethylene production, the standard deviation ranged from 0.24 for 50 SNPs to 0.06 for 61,030 SNPs. Furthermore, it should be noted that traits with a rather simple genetic architecture due to the contribution of major QTL to the phenotypic variation such as Ethylene, RI as well as Citric.A were the most sensitive to the variation of the number of markers.

### Optimization of genomic prediction

#### Optimization of the GS models:

**Figure 5:**
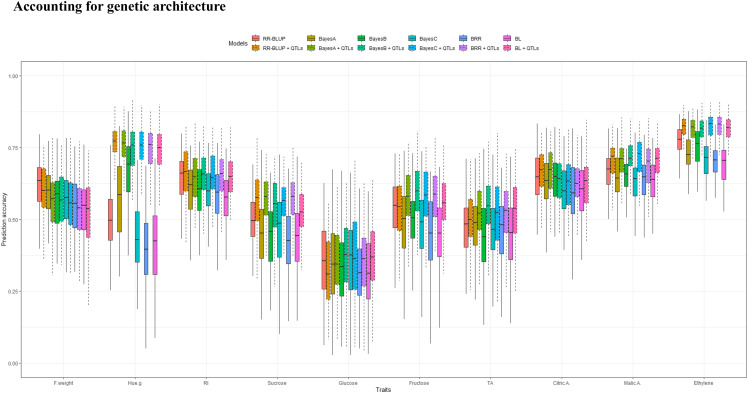
Accuracy of genomic prediction for 10 apricot fruit quality traits using six statistical models treating all SNPs as random effects (boxplots with solid line type) and models where SNPs, significantly linked to apricot quality traits within randomly drawn training partitions, were considered as fixed effects (boxplots with dashed line type). Prediction accuracies were computed using a random cross-validation scheme replicated 100 times.

We investigated the effect of integrating prior knowledge about the trait genetic architecture on the accuracy of genomic prediction. The prediction performance of models in response to the inclusion of QTL significantly linked to the traits of interest varied across models and traits (Figure 5). This optimization approach resulted in a consistent gain in accuracy for all traits with the exception of F.weight. Accuracy gain derived from models with QTL as fixed effects in comparison with models with markers as random effects was more pronounced for Hue.g for which adding two QTL explaining a substantial proportion of phenotypic variance resulted in an accuracy gain of 25.8% across the six investigated models. Similarly, for ethylene production, upweighting major QTL improved accuracy by 9.4%. The magnitude of gain in PA was tightly linked to the proportion of variance explained by QTL added to prediction models with R^2^ = 0.67. Furthermore, models built with regard to trait genetic architecture also permitted to enhance PA for sugars and organic acids, for which gains ranged from 2.3 to 10.5%. Nevertheless, the integration of QTL as fixed effects in the models slightly decreased the prediction performance for F.weight, with decreases ranging from -2.1% to -0.1%. Furthermore, the prediction models differed in their response to the integration of prior genomic information. The gain in PA ranged from 4.6 to 10.3%. The highest gain was observed for the BRR model, although RR-BLUP depicted the lowest increase in PA.

#### Multi-trait genomic prediction:

**Figure 6:**
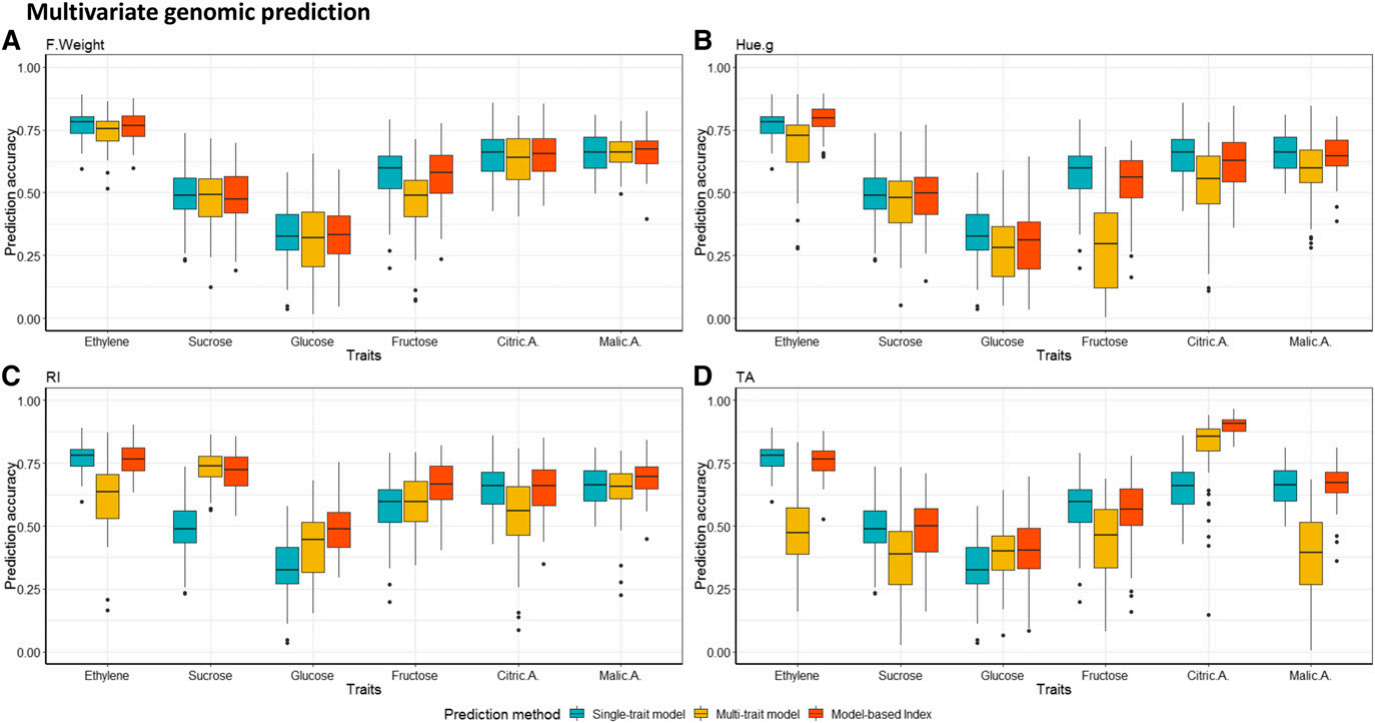
Assessment of multivariate genomic prediction of ethylene production, content in organic acids (malic acid and citric acid) and soluble sugars (sucrose, glucose and fructose) using F.weight (A), Hue.g (B), RI (C), and TA (D) as proxy predictands in comparison to univariate prediction. Multivariate models leveraged the information provided by secondary traits using either phenotypic values (multi-trait model) or genomic estimated breeding values (model-based index).

In order to improve the accuracy of genomic prediction, we assessed the univariate prediction in comparison with multivariate prediction, by leveraging the information on secondary traits that are easy-to-measure, such as F.weight, Hue.g, RI and TA, in order to predict the ethylene production rate and the apricot fruit content in organic acids (Malic.A and Citric.A) and in soluble sugars (Sucrose, Glucose and Fructose) (Figure 6). Multi-trait prediction using genetic values yielded improvement in PA notably for traits which showed high positive pairwise genetic correlations such as sugars and RI, Citric.A and TA, ethylene and Hue.g. By contrast, model-based index was approximately equivalent to univariate models for non-correlated traits (Figure 6A). Nevertheless, multivariate models using phenotypic information on secondary traits to predict focal traits displayed the lowest overall PA for all the traits under investigation (Figure 6). Prediction performance for ethylene production, organic acids and sugars was significantly improved subsequent to the integration of estimated genetic values of Hue.g, RI and TA (Figures 6B, 6C and 6D, respectively). The model-based index showed a gain in accuracy which reached 0.25 for Citric.A. informed by TA (Figure 6D), 0.23 for sucrose content informed by RI, 0.17 for glucose informed by RI, and 0.08 for fructose informed by RI (Figure 6C). Nevertheless, the model-based selection index displayed a slight drop in accuracy when genetic correlation between secondary traits and focal traits was low. The decrease ranged from 0.01 to 0.04 for traits where genetic correlation ranged from -0.86 to 0.24.

**Figure 7:**
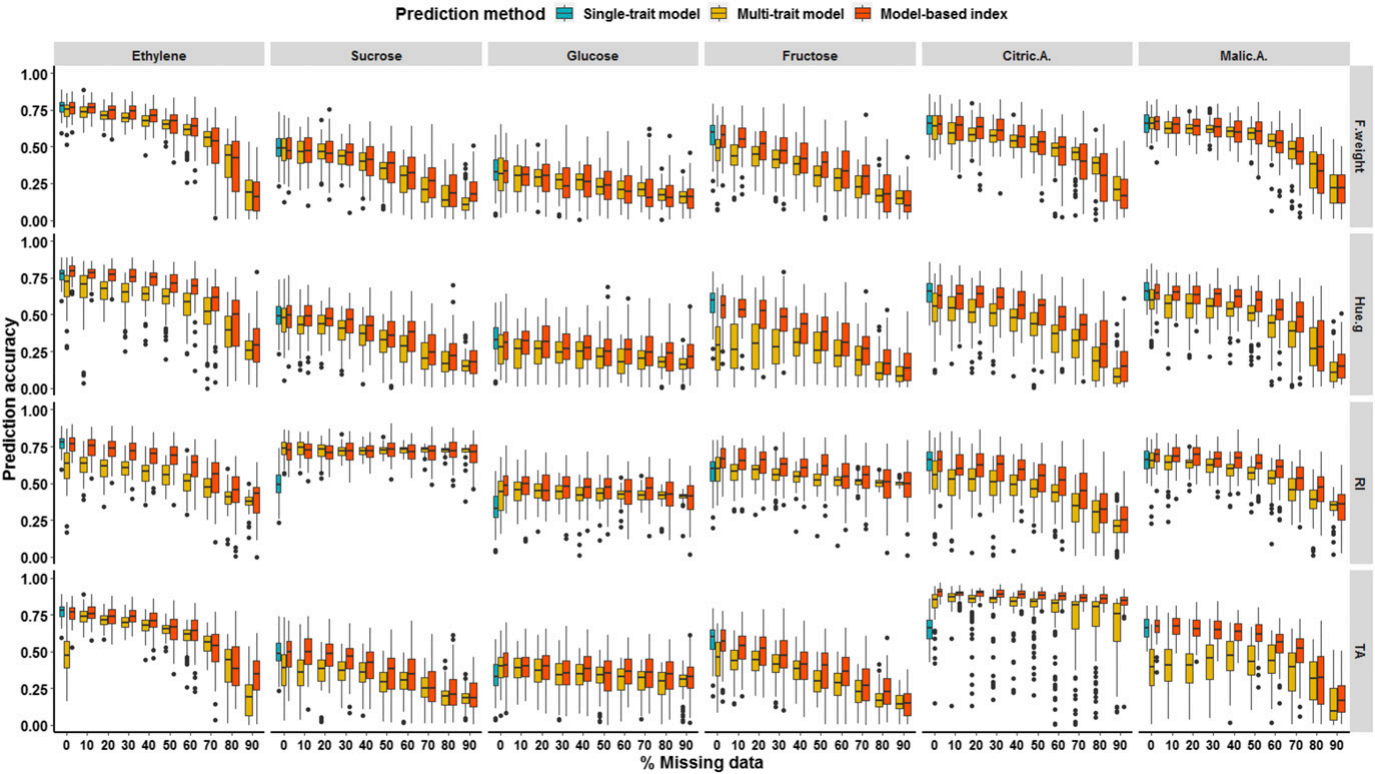
Evaluation of the multi-trait genomic prediction framework where missing values included within the training partition, with an increasing missing rate ranging from 0 to 90% of focal traits (Ethylene, Sucrose, Glucose, Fructose, Citric.A. and Malic.A.) predicted using proxy traits (F.Weight, Hue.G, RI and TA). Multi-trait strategy was modeled using either phenotypic values (multi-trait model) or genomic estimated breeding values (model-based index) in comparison with single-trait GBLUP model.

Further, as depicted in Figure 7, the inclusion of an increasing percentage of missing data for target traits within the training partition resulted in a decrease in PA for all the traits in comparison with bivariate models that did not include missing values. However, bivariate models with 90% of missing data outperformed single-trait models with 0% of added missing values for Sucrose and Glucose informed by RI and for Citric.A informed by TA that yielded strong genetic correlation and for which models were built using only 11 individuals. Interestingly, bivariate predictions that modeled jointly Glucose and TA, with 90% of missing values, exhibited higher accuracy compared to univariate models despite their strong negative correlation (-0.72). Average gain in accuracy derived from bivariate models conceived with only 10% of the training partition ranged from 2.2% for (Glucose, RI) to 20.5% for (Sucrose, RI). In addition, the model-based index strategy yielded higher accuracies with respect to bivariate predictions that model phenotypic values rather than GEBVs of proxy traits.

## Discussion

The objective of our study aimed at investigating GS within a biparental cross between two apricot varieties Goldrich and Moniqui contrasting for fruit traits. The emphasis on GS in apricot breeding stems from the need to deliver genetic progress by facing constraints that hamper or slow down genetic improvement in this species such as the length of juvenility period and the complex genetic architecture of several traits of interest. In this context, our work represents the first evaluation of GS in apricot with a focus on key fruit quality attributes in order to help selection decisions within quality-oriented breeding schemes. Moreover, our study provided new insights into the genetic architecture of fruit quality parameters for fruit weight, ethylene production, and the content in soluble sugars and organic acids. The optimization of genomic PA was also explored by including QTL significantly linked to targeted traits in prediction models and within a multivariate prediction framework.

### Exploration of the phenotypic data

Exploring the phenotypic data revealed that fruit Hue.g and ethylene production showed skewed distributions, which feeds to the concomitant existence of orange skinned fruits with low ethylene production and white colored fruits with high ethylene outburst, reflecting the scope of variation of fruit quality traits within the Go×Mo progeny. Nevertheless, fruit weight and contents in glucose and fructose present symmetric distributions coupled with low standard deviation. A particular variability was found for the sucrose content, the main soluble carbohydrate derived from photosynthesis, which accounts for 60–80% of total soluble sugars stored in the apricot fruit. The related variability could result from source-sink relationships. Indeed, carbohydrate translocation from source leaves to ‘non-photosynthetic’ sink organs, notably fruits, depends largely on agricultural practices such as fruit thinning which tends to increase the availability of photo-assimilates in the remaining fruits (Roussos *et al.* 2011). Therefore, as no thinning was performed in the Go×Mo progeny, this likely resulted in a stronger competition for carbohydrates between sink organs thereby affecting fruit weight and carbohydrate concentration over the two years of observation. In addition, a broad range of variability was mirrored in Hue.g within the Go×Mo population, which originates from color-contrasted varieties, with a variation magnitude that ranges from deep-orange to white. Indeed, the fruits of Goldrich tend to accumulate colored carotenoids (β-carotene) while those of Moniqui mainly accumulate uncolored compounds notably phytoene and phytofluene (Marty *et al.* 2005; Adami *et al.* 2013; Jiang *et al.* 2019). Likewise, Goldrich and Moniqui show different climacteric behaviors with regard to ethylene production that ranges from fruits with a high ethylene production rate and fast evolution toward maturity (Moniqui), to fruits characterized by lower ethylene production (Goldrich). With respect to fruit organic acidity, the Go×Mo progeny exhibited a wide range of phenotypic variability for TA (from 11.3 to 41 meq 100g^-1^), citric acid (from 8.0 to 45.3 meq 100g^-1^) and malic acid (from 4.1 to 17.9 meq 100g^-1^). The citric to malic acid ratio ranged from 0.9 to 8.5 with a predominance of citric acid in Go×Mo. These results are in accordance with previous studies expressing large genetic diversity in apricot germplasm (Gurrieri *et al.* 2001; Bassi and Audergon 2006).

It is noteworthy that the genotype effect contributes significantly to the variability of target traits within the Go×Mo population. Therefore, the phenotypic variation linked to apricot fruit quality is largely due to genetic differences and thus the contribution of genetic pattern to the overall phenotypic variance, which is confirmed by estimation of trait heritability. Indeed, except for the sucrose content, all the apricot fruit quality traits were highly heritable. Moreover, the quality attributes varied in response to the fruit physiological stage, the year effect and the genotype × year interaction. Hence, components of phenotypic variance resulted from an interplay of several factors including both genetic and environmental conditions. However, the phenotypic variation differed between traits. For instance, climatic fluctuations tended to exert minor effect on sucrose, glucose and fructose contents, although genotype by year interaction highly contributes to the expression of sugar-related traits. Contrastingly, acid-related traits (TA, contents in citric and malic acids) were considerably dependent on the year effect. This trend has been confirmed by several studies, which highlighted that a higher acidity pertains to fruits that were produced under cold and wet weather while fruits with low acid flavor are produced under warmer temperatures (Wang and Camp 2000; Gautier *et al.* 2005; Etienne *et al.* 2013).

### QTL detection

The genetic architecture underlying the fruit quality attributes has been assessed in order to deepen our knowledge of the genomic regions harboring QTL for apricot quality. The exploration of genetic determinism of target traits revealed highly stable QTL across the two years for all traits under consideration, except for sucrose and glucose contents. Several QTL were identified at the same position on the two parental maps for eight traits out of 10, except for the contents in glucose and citric acid, which suggests the stability of these QTL over genetic backgrounds. Three QTL clusters were identified: (i) ethylene, fructose and fruit weight on LG1 of Goldrich, (ii) ethylene, TA, citric acid and malic acid on LG2 of Goldrich and (iii) ethylene, RI, fructose, sucrose, on one hand, malic acid and Hue.g on the other hand on LG2 on Moniqui. QTL clusters might arise from pleiotropic effects of one QTL or the presence of tightly linked QTL (Eduardo *et al.* 2011). QTL linked to ethylene production and acid-related traits which clustered on LG2 on Goldrich as well as QTL clustered for sugar-related traits, organic acids and ethylene on LG2 Moniqui underline overlapping patterns in the expression of apricot quality attributes, notably for ethylene. Indeed, ethylene is a phytohormone interfering in several metabolic pathways and whose biosynthesis is coupled with a respiratory burst. Linked with subsequent ethylene signal and related transcription factors, several physical and biochemical changes occur in fruit maturation such as chlorophyll degradation, carotenoid accumulation and modulation of sugar content, as well as changes in organic acids profiles (Paul *et al.* 2012). Additionally, QTL colocalization between ethylene production and organic acids content in the Go×Mo progeny, confirmed by significant phenotypic correlations, demonstrates that these traits are likely to segregate together so that the fruits whose ethylene production is high, can also produce large amounts of citric acid and low amounts of malic acid. Our results support the causality link between ethylene production and organic acids, which is consistent with several studies postulating that metabolic pools of citric and malic acids are under ethylene regulation (Fan *et al.* 1999; Defilippi *et al.* 2004; Gao *et al.* 2007; Valdés *et al.* 2009; Etienne *et al.* 2013; Batista-Silva *et al.* 2018).

A major and robust QTL was mapped for Hue.g, located on the LG3 of Goldrich supporting results of Socquet-Juglard *et al.* (2013). Similarly, different studies on *Prunus* species have also reported that LG3 is associated with skin and flesh pigmentation in conjunction with carotenoid and anthocyanin contents (Frett *et al.* 2014; Salazar *et al.* 2017; García-Gómez *et al.* 2019). Further, a cluster including QTL for ethylene and Hue.g was only detected for Moniqui. This is in adequacy with the results of Marty *et al.* (2005) according to which the synthesis of colorless carotenoid precursors, phytoene and phytofluene, is upregulated by ethylene, while β-carotene, the main colored carotenoid pigment, is ethylene-independent. This trend has been confirmed by a steeper expression of carotenogenic genes in white fruits in comparison to orange ones (Marty *et al.* 2005).

### Genomic prediction accuracy

Our investigation of the potential of GS for apricot fruit quality revealed that within-family predictions hold a great promise since accuracies varied across traits within a range from 0.31 to 0.78. Our results are in accordance with Riedelsheimer *et al.* (2013) who evidenced the need to train prediction models using full-sibs for the validation set. Hence, cross-validation performed within-family provides richer information than that issued from distant relatives, given the identity-by-descent (IBD) relationships among full-sibs (Legarra *et al.* 2008). Therefore, within-population prediction presents a valuable tool for the implementation of genomic prediction. It allows to achieve higher genomic PA than predictions across multiple populations (de Roos *et al.* 2009). However, despite its robustness and stability, a mainstream pitfall encountered in within-population genomic prediction lies in its limited flexibility and thus the need for expanding predictions to multi-population training sets (Schopp *et al.* 2017).

Moreover, for a full-sib family, the high level of relatedness and the strong LD between SNPs and causal QTL underlying the traits under investigation result in high PA. Our results are in accordance with previous studies that pointed out the importance of the inclusion of relatedness in the prediction model (Lenz *et al.* 2017). Interestingly, the highest PA were obtained for ethylene production rate, a trait whose direct measurement is time-consuming, making it unsuitable for high-throughput studies. Conversely, the lowest PA was found for the fruit content in sucrose, glucose and fructose. This might be attributed to the non-genetic part of the observed variation due to environmental factors. Indeed, postharvest performance strongly depends upon various preharvest factors that modulate the source-sink relationships. It is noteworthy that the fruit sweetness has a tendency to increase in response to cultural practices such as fruit thinning. As sucrose is prominent metabolites in the photosynthetic carbon scheme, and as the increase in availability of this carbohydrate is considerably dependent upon fruit load during the secondary fruit growth phase (Roussos *et al.* 2011), the lack of precise source-sink control by thinning strongly impacted the quality of the prediction.

### Factors controlling genomic prediction accuracy

#### Impact of statistical prediction models:

Across all prediction models, the average accuracy for apricot quality traits were moderate to high. This trend is in adequacy with the extent of linkage disequilibrium (LD) between SNPs and QTL. Hence, in a single generation cross, as a limited number of recombination events occurs per meiosis, leading to large linkage blocks and therefore, more phenotypic records per chromosome segment are available in order to derive GEBVs which leads to more accurate predictions (Lorenzana and Bernardo 2009). Within the framework of model comparison, RR-BLUP tended to outperform Bayes A, Bayes B, Bayes C, BL and BRR for six traits out of 10. RR-BLUP proved to be the best-performing statistical model notably for traits controlled by several QTL that explain each a small amount of the phenotypic variance. In addition, RR-BLUP is more efficient with regards its computational speed in comparison to Bayesian models (Tan *et al.* 2017). Bayes B showed a superior prediction performance compared to RR-BLUP for Hue.g where a QTL accounts for a large proportion of the phenotypic variation. The aforementioned outcome is in agreement with (Daetwyler *et al.* 2010), in which Bayes B gave higher accuracies than GBLUP when the number of QTL $N_{QTL}$ was low. However, this trend diminished as $N_{QTL}$ increased past the equivalence point where $N_{QTL}$ equates to independently segregating chromosome segments$M_{e}$. The deviation from superiority of RR-BLUP for oligogenic traits is likely due to the model over-parameterization as a response to fitting a large number of SNPs to model variation within a trait controlled by few major QTL (Resende *et al.* 2012b). Moreover, the performance of RR-BLUP is due to genetic relatedness captured by markers, due to a higher proportion of shared alleles between full-sibs as compared to genetically distant individuals within the training set (Habier *et al.* 2007). Further, a study driven by Heslot *et al.* (2012) focused on comparison of GS models revealed that if predictive performance of a given model is mainly grounded on kinship such as RR-BLUP, accuracy decreases much faster in comparison to models based on LD between SNPs and QTL such as Bayesian approaches. This trend is due to an increase in inbreeding at a faster pace for kinship-based models compared to LD-based models, owing to the decay of genetic relationships. Therefore, RR-BLUP is not recommended to approximate marker effects since the contribution of genetic relationships to the prediction performance is halved each generation. Conversely, accuracy due to LD pattern is more persistent (Habier *et al.* 2007).

#### Impact of training population size:

Lowering the TP size led to a decay in PA. Accordingly, further improvement on PA could be attained by increasing the total training population size, as a larger reference set provides more acurate predictions due to less biased estimation of marker effects. Furthermore, besides the prominent effect of the size of training set, the design of the reference population in respect of resemblance between training and validation partitions, depicts a potent factor that considerably affects prediction performance. Thereby, closer relationships between training and validation populations has been reported to lead to a higher PA. Conversely, adding genetic diversity within the reference population lead to a reduction in PA compared with smaller training populations including highly related individuals (Riedelsheimer *et al.* 2013; Lehermeier *et al.* 2014; Lorenz and Smith 2015). More importantly, highly related individuals share long haplotypes and linkage blocks due to limited recombination events and thereby lead to minor bias while computing GEBVs within the validation set (Lorenzana and Bernardo 2009; Hickey *et al.* 2014; Lozada *et al.* 2019). Hence, higher accuracies linked to richer information issued from closely related individuals rather than distant individuals arise from more precise estimation of marker effects. Therefore, higher degree of IBD sharing between full-sibs is likely to provide accurate estimation of genetic variation for quantitative traits exempt from confounding non-genetic factors in comparison to genetically distant individuals (Visscher *et al.* 2006).

#### Impact of the number of markers:

The number of markers used to train prediction equation represents a prominent factor that affects the prediction performance. Hence, the larger the set of markers the higher the probability to be in LD with QTL controlling target traits, which provided richer genomic information. This trend has been shown by de los Campos *et al.* (2013) who highlighted that the inclusion of all available markers resulted in a considerable increase of the proportion of variance explained. Thereby on a broader scope, the number of SNPs required to obtain accurate predictions depends on the number of independently segregating chromosome segments $M_{e}$ as well as the span of LD within the study population (Goddard 2009; Daetwyler *et al.* 2010). Nevertheless, in the present study, the PA tended to reach a plateau for 6,103 SNPs, indicating that only 10% of the initial markers set were sufficient to capture SNPs-QTL LD, related to several traits. Our results are consistent with those of Covarrubias-Pazaran *et al.* (2018) who showed that a medium marker density (500 to 750 SNPs) was sufficient to achieve high accuracy due to extensive LD typically present in biparental populations. Similarly, phenotypic records collected in maize biparental populations that were closely related to the selection candidates, only a small number of SNPs (200 – 500) and relatively small number of phenotypes (1,000) were needed to achieve accurate predictions of GEBVs. Otherwise, in more distantly related populations, 10,000 SNPs as well as 5,000 to 20,000 records are needed (Hickey *et al.* 2014). In our study, no accuracy gain was noted beyond 6,103 SNPs. Similarly, Hickey *et al.* (2014) reported no benefit in terms of PA beyond 10,000 SNPs. Further, in an Eucalyptus breeding population of 949 F1 hybrids, no significant accuracy improvement was obtained using more than 5,000 SNPs to predict growth and wood traits (Tan *et al.* 2017). In this latter study, only 500 to 1,000 informative, non-redundant and randomly distributed markers were needed to reach sufficient coverage of the genome (Tan *et al.* 2017). More importantly, our study showed that higher marker density can lead to a reduction in PA whatever the trait. This decrease in accuracy might be attributed to multicollinearity between SNPs due to overfitted prediction models, overestimating the marker effects (Meuwissen *et al.* 2001; Muir 2007).

### Genomic prediction optimization

#### Optimization of the GS models:

The genomic prediction including QTL mapping outcomes considerably depends on the genetic architecture of the traits under consideration. Hence, this study showed that the magnitude of accuracy gain in prediction was heterogeneous across the traits studied. For instance, for Hue.g, a significantly higher accuracy was obtained as a result of the inclusion of two QTL that represent more than 58% of phenotypic variation, compared to models where all markers were fit as random effects. Similar patterns were observed for RI and contents in sucrose and fructose due to the large proportion of genetic variance captured by fixed factors in the prediction models. Hence, accounting for prior genomic information provided a steeper increase in accuracy for traits controlled by major QTL such as Hue.g, fructose, sucrose and RI compared to the other fruit quality metrics controlled by several QTL explaining lower proportion of phenotypic variance. Our findings are in agreement with those of Morgante *et al.* (2018), who showed that the integration of *a priori* information on the genetic architecture underlying quantitative trait variation resulted in a valuable increase in accuracy within samples of unrelated individuals. Similarly, Zhang *et al.* (2014) reported that GBLUP informed by the genetic architecture in rice diversity panel an increased the accuracy by 5.4%. However, when QTL were modeled as fixed effects in models, a slight decrease in prediction for fruit weight, glucose and TA was observed, since these traits are controlled by QTL covering only a small proportion of variation. An additional feature of these QTL was their instability across years.

#### Multi-trait genomic prediction:

Within the multivariate genomic predictions, our findings highlighted that prediction models targeting multiple traits are greatly dependent upon genetic correlations. Thereby, we showed that multivariate models generally provided more accurate predictions compared to univariate models for genetically highly correlated traits. Nevertheless, accuracies showed an equivalent or slight decrease under a low genetic correlation framework. Similar outcomes were reported by several studies. For instance, in bread wheat, Michel *et al.* (2018) exploited the availability of easy-to-measure traits such as the protein content, which is genetically highly correlated with costly and labor-intensive traits linked to baking quality in order to breed for superior genotypes. In addition, our results are in accordance with those of Calus and Veerkamp (2011) where the accuracy gain ranged from 0.03 to 0.14, when genetic correlation of target traits varied from 0.25 to 0.75. However, multivariate models grounded on phenotypic values performed poorly in comparison to model-based index which accounted for estimated genetic values despite phenotypic information. An exception was noted for the prediction of sucrose content informed by RI, where multi-trait model outperformed the two previously cited models, given that genetic correlation between sucrose and RI is very close to 1, so that the residual correlation between these two traits is almost null. Besides their superiority with respect to prediction performance, model-based selection index are computationally less demanding than phenotype-based multivariate models (Michel *et al.* 2018). More importantly, the accuracy gain is more pronounced for slightly heritable traits that are genetically correlated with a highly heritable trait such as sucrose content (H^2^ = 0.56), which is highly genetically correlated to RI (Jia and Jannink 2012; Guo *et al.* 2014; Karaman *et al.* 2018). Furthermore, the drop-off in PA for traits that are not genetically correlated is attributed to the residual correlation which potentially adds noise to predictions with respect to single trait models and thus leads to biased computation of GEBVs. Our results are in agreement with Covarrubias-Pazaran *et al.* (2018), which showed no benefit over single-trait models under a low genetic correlation framework. Therefore, broad phenotypic information provided at high-throughput on easy-to-measure traits such as F.weight, Hue.g, RI and TA could offer the opportunity to enlarge the selection candidate population. This potentially enhance the PA for costly and labor-intensive traits. Hence, multivariate prediction grounded on easy-to-phenotype traits might help selection decisions and thus potentially deliver genetic progress notably for perennial species for which the length of breeding cycles is a significant impediment to genetic improvement process.

## Conclusion

Our findings highlighted that GS holds a valuable potential with reference to prediction of fruit quality within a biparental design in apricot. Indeed, genomic prediction yielded interesting outcomes in terms of PA which encourages further investigations about valuing whole-genome information with the aim of assessing agronomical relevant traits in apricot and potentially orienting strategies toward the implementation of GS within breeding schemes. Furthermore, the outcomes of this study provided insights into the genetic architecture of apricot fruit quality whose integration in prediction models led to a higher PA. As expected, PA gain is higher for the traits that are governed by QTL explaining a substantial part of phenotypic variation. Besides, genomic predictions might be improved by optimizing factors controlling the predictive performance of GS models such as larger training populations, for example. However, with reference to markers’ density, only 6,103 SNPs were enough to reach accurate predictions. In terms of prediction modeling, RR-BLUP outperformed Bayesian models and provided a valuable compromise between statistical performance and computational time. Moreover, optimal accuracies were obtained under a multivariate prediction framework for fruit quality traits that are strongly and positively correlated to their different proxies, and thus predictions of ethylene content informed by Hue.g, organic acids by TA and sugars by RI are more accurate than univariate predictions.

In terms of prospects, regarding that the response of phenotypes to genomic prediction is tightly linked to the observed variability within the study population, a greater attention should be paid to orchard management practices through mastering source-sink relationships in order to optimize the potential performance of genotypes. In addition to that, in the present study, conclusions on the efficiency of GS in apricot were drawn for a biparental design in which genotypes share the same LD pattern and relatedness between training partition and validation partition is high. Therefore, further evidence ought to be assessed in a genetic diversity panel potentially covering a broader range of allelic combinations of traits of agronomical interest.
